# Enantio-sensitive unidirectional light bending

**DOI:** 10.1038/s41467-021-24118-4

**Published:** 2021-06-24

**Authors:** David Ayuso, Andres F. Ordonez, Piero Decleva, Misha Ivanov, Olga Smirnova

**Affiliations:** 1 Max-Born-Institut, Berlin, Germany; 2 Department of Physics, Imperial College London, London, UK; 3 Technische Universität Berlin, Berlin, Germany; 4 Dipartimento di Scienze Chimiche e Farmaceutiche, Università degli Studi di Trieste, Trieste, Italy; 5 Institut für Physik, Humboldt-Universität zu Berlin, Berlin, Germany

**Keywords:** High-harmonic generation, Ultrafast photonics, Attosecond science

## Abstract

Structured light, which exhibits nontrivial intensity, phase, and polarization patterns in space, has key applications ranging from imaging and 3D micromanipulation to classical and quantum communication. However, to date, its application to molecular chirality has been limited by the weakness of magnetic interactions. Here we structure light’s local handedness in space to introduce and realize an enantio-sensitive interferometer for efficient chiral recognition without magnetic interactions, which can be seen as an enantio-sensitive version of Young’s double slit experiment. Upon interaction with isotropic chiral media, such chirality-structured light effectively creates chiral emitters of opposite handedness, located at different positions in space. We show that if the distribution of light’s handedness breaks left-right symmetry, the interference of these chiral emitters leads to unidirectional bending of the emitted light, in opposite directions in media of opposite handedness, even if the number of the left-handed and right-handed emitters excited in the medium is exactly the same. Our work introduces the concepts of polarization of chirality and chirality-polarized light, exposes the immense potential of sculpting light’s local chirality, and offers novel opportunities for efficient chiral discrimination, enantio-sensitive optical molecular fingerprinting and imaging on ultrafast time scales.

## Abstract (web summary)

Developing new methods for structuring light’s chirality in space would be advantageous for various next-generation applications. Here, the authors report enantio-sensitive unidirectional light bending by interacting light with isotropic chiral media.

## Introduction

Chirality, or handedness, is a ubiquitous geometrical property found in both light and matter. Mirror reflection transforms a chiral object into its opposite counterpart, with our left and right hands being a typical example. These mirror twins are called enantiomers, and symmetry dictates that they behave identically unless interacting with another chiral object. Chirality is of tremendous importance in nature and distinguishing molecular enantiomers is vital, stimulating major research efforts aimed at increasing the efficiency of enantio-discrimination by relying on effects that occur within the electric-dipole approximation, see e.g.^1–12^.

Here we describe a new highly enantio-sensitive phenomenon, which relies on structuring light’s handedness in time and space, to effectively control the handedness of local emitters in the chiral medium, complementing the rich family of vectorial light structures^13–25^. It allows us to realize a chiral version of Young’s double slit experiment, which ‘bends’ the non-linear optical response of a chiral medium in an enantio-sensitive and molecule-specific manner.

## Results

### The chiral double-slit Gedankenexperiment and polarization of chirality

**Fig. 1:**
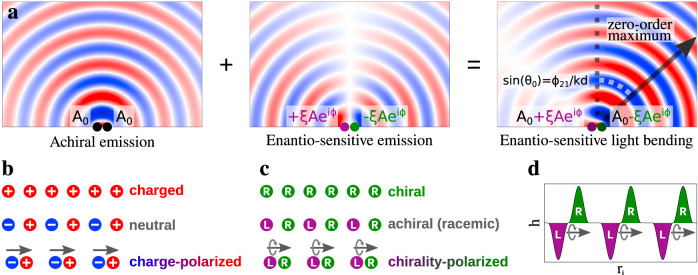
Chiral Young’s double slit Gedankenexperiment. **a** The superposition of the achiral component of the emission (even with respect to the center, left panel) and the enantio-sensitive component of emission (odd, central panel) leads to enantio-sensitive bending of the emitted light (right panel). **b** Mono-dimensional (1D) arrangement of charged units that is: charged and unpolarized, neutral and unpolarized, and neutral and polarized. **c** 1D arrangement of chiral units that is: chiral and unpolarized, achiral and unpolarized, and achiral and polarized. **d** Sketch of the handedness of a light field that is chirality-polarized along the spatial coordinate *r*_*i*_.

The chiral Young’s double-slit experiment involves two chiral emitters of opposite handedness at points **r**_**1**_ and **r**_**2**_ separated by a distance *d* = ∣**r**_**1**_ − **r**_**2**_∣. Since chiral emitters of opposite handedness are characterized by fields of equal amplitude emitted out of phase^4,8^, the emitted fields at these two points are: **P**_1_ = (**A**_**0**_ + *ξ***A***e*^*iϕ*^)*e*^−*iωt*^ and **P**_2_ = (**A**_**0**_ − *ξ***A***e*^*iϕ*^)*e*^−*iωt*^, where **A**_**0**_ is a common non-chiral sensitive component of the emitted field, **A** is the amplitude of each chiral-sensitive component, *ϕ* is the phase delay between the chiral and achiral components, and *ξ* = ±1 defines the handedness of the slit at position **r**_**1**_. The interference term defining the position of maxima and minima of the interference pattern (Fig. 1a) is proportional to $\cos (kd\sin \theta -{\phi }_{21})$, where *ϕ*_21_ is the relative phase between the two slits, and yields the following expression for the position of the interference maximum of *m*-th order:

1 $$kd\sin {\theta }_{m}={\phi }_{21}+2\pi m,\tan {\phi }_{21}=\frac{2\xi {\bf{A}}\cdot {{\bf{A}}}_{{\bf{0}}}\sin \phi }{{{\bf{A}}}^{2}-{{\bf{A}}}_{{\bf{0}}}^{2}}.$$

Eq. (1) shows that the interference pattern is shifted to the right or to the left, depending on the sign of *ξ*. Note that since the two chiral emitters have opposite handedness, the overall medium, which realizes this double slit experiment, is achiral. However, the spatial arrangement of the two chiral slits breaks the parity symmetry of the interference pattern. Indeed, the achiral component of the emission coming from the two slits is an *even* function of coordinates, while the enantio-sensitive component of the emission from the two slits is an *odd* function of coordinates. Superposition of these even and odd functions breaks the parity symmetry, leading to unidirectional deflection of the emitted light described below. Importantly, the way parity is broken, i.e. whether the first slit is left-handed and the second slit is right-handed, or vice versa, defines the direction of light deflection. To characterize the spatial arrangements of chiral slits in an overall achiral medium, we introduce the concepts of *polarization of chirality* and *chirality dipole*.

Figure 1b,c illustrate the concepts of polarization of chirality and the chirality dipole, in analogy with the polarization of charge. Figure 1b shows a one-dimensional arrangement of alternating positive and negative charges ±*q*. When the charges are uniformly distributed, the medium is not polarized. It becomes polarized as we modify their positions, creating dipoles **d**_*e*_ = *q***r**_0_, where **r**_0_ is the vector connecting the nearby negative and positive charges. Consider now a similar racemic distribution of chiral emitters, or other chiral units, of alternating handedness, Fig. 1c. Just like the neutral medium of charged particles, this racemic distribution is unpolarized if the distances between consecutive chiral units are the same. If we modify them, e.g. by shifting the right-handed units to the left, we create *dipoles of chirality*, and the medium acquires *polarization of chirality*.

The chirality-polarized chain of alternating left- and right-handed emitters in Fig. 1c constitutes a multi-slit version of the chiral Young’s double slit experiment. Symmetry dictates that the spatial dependence of the achiral component of the emitted light field should follow an even periodic function of *x*, e.g. $G(x){A}_{0}\cos (\kappa x)$, while the chiral component should follow an odd periodic function, e.g. $\xi G(x)A{e}^{i\phi }\sin (\kappa x)$, where *x* is a coordinate along the line connecting the slits and the envelope ∣*G*(*x*)∣^2^ describes the intensity distribution of emitters in the linear chain. The interference of these even and odd contributions in *k*-space shifts the interference pattern toward negative or positive *k*-vectors (see Methods for further details). That is, the emitted light will bend to the left or to the right from the center of the envelope ∣*G*(*x*)∣^2^, depending on the sequence in which the chiral slits alternate, encoded in the sign of *ξ*. Thus, even though the total amount of left-handed and right-handed emitters under the envelope is the same, their interference leads to the enantio-sensitive outcome, just like its double slit version.

One way of creating chiral emitters with controlled handedness and positions is to structure the handedness of locally chiral light fields to engineer the chirality dipoles introduced above. Light’s local handedness *h*(**r**) is determined by the handedness of the chiral Lissajous figure drawn by the tip of its electric field vector **F**(*t*) in time at any point **r**. It can be quantified via the chiral correlation functions introduced in^26^ (see Methods).

Suppose that light’s *h*(**r**) changes sign from one point to another, so that it is left-handed at one point, right-handed at another, and achiral in between (see Fig. 1d). Such spatial structuring of *h*(**r**) imparts phase and amplitude gratings onto the nonlinear optical response of chiral molecules of a given handedness. As a result, the nonlinear response of isotropic chiral matter, e.g. randomly oriented chiral molecules, captures the spatial distribution of the field’s handedness and realizes the desired sequence of chiral emitters (slits).

The chiral-sensitive components of the fields emitted by neighbouring slits are out of phase with each other, while the relative phase between the chiral and achiral emission components are determined by the molecule and the structured light. Moreover, such phase profile will “bend” the emitted light, shifting the interference pattern to the left or to the right, depending on the molecular handedness. Indeed, for the same *h*(**r**), a medium of randomly oriented molecules of opposite handedness will realize opposite sequences of emitters: if left molecular enantiomers generate the sequence *RL* *RL* *RL*..., then right molecular enantiomers generate the sequence *LR* *LR* *LR*...

### Formal approach

A formal approach (see Methods) allows us to quantify the enantio-sensitive light bending, the chirality dipoles, as well as the more complex spatial distributions of chirality and their consequences. Fundamentally, the chirality dipole and higher order chirality multipoles characterize the distributed handedness of a racemic object, with the chirality dipole being its first moment. In our case the racemic object is the driving field. Its interaction with homogeneous chiral media results in a non-linear far-field response that records the structure of the driving field in **k**-space (rather than in **r**-space). Accordingly, the distributed handedness encoded in the far-field response is the one with respect to **k**-space. Indeed, the first and higher-order moments of the **k**-space handedness $\tilde{h}({\bf{k}})$, namely

2 $$\tilde{{\bf{h}}}=\int {d}^{3}k\,\, {\bf{k}}\tilde{h}({\bf{k}}),\,\,\,{\tilde{h}}_{ij...}=\int {d}^{3}k\,\, {k}_{i}{k}_{j}...\tilde{h}({\bf{k}}),$$

describe the enantio-sensitive part of the far-field intensity (see Methods). Note that the zeroth momentum,

3 $${h}_{0}=\int {d}^{3}k\ \tilde{h}({\bf{k}}),$$

which describes the enantio-sensitive contribution to the integrated light intensity, is equal to zero for a racemic object. But even if *h*_0_ = 0, enantio-sensitive effects remain. For example, if light is racemic, *h*_0_ = 0, but chirality polarized, i.e. $\tilde{{\bf{h}}}\;\ne\; 0$, we will see enantio-sensitive unidirectional deflection in the nonlinear optical response.

**Fig. 2:**
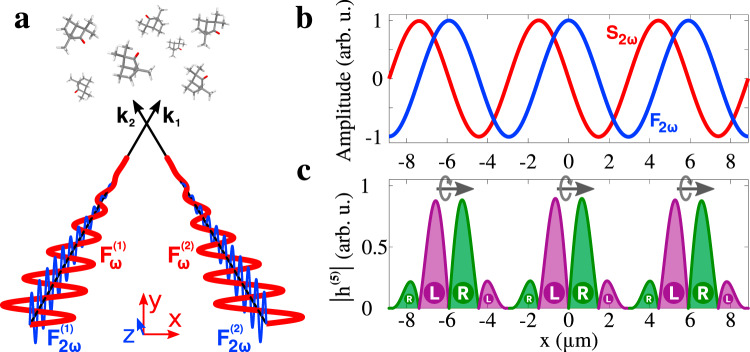
Chirality polarized, but racemic, light. **a** Two non-collinear beams carry an *ω* field, linearly polarized in the *xy* propagation plane, and an orthogonally polarized 2*ω* field, with the same two-color delay in both beams. **b** Normalized 2*ω*-field amplitude (*F*_2*ω*_, blue line) and transverse spin (*S*_2*ω*_ ∝ *F*_*x*_*F*_*y*_, red line). **c** Local handedness of the light field, characterized by its fifth-order chiral correlation function *h*^(5)^. The colors encode the phase of *h*^(5)^ and thus the field’s handedness, which is controlled by the relative phase *ϕ* (see Eq. (4) and Methods); purple: $\arg \{{h}^{(5)}\}=2\phi +0.5\pi$, green: $\arg \{{h}^{(5)}\}=2\phi -0.5\pi$. Since the chirality integrated over x is equal to zero, the light is racemic. The gray arrows indicate the direction of polarization of chirality. We have used the following laser parameters: *ω* = 0.044a.u. (*λ* = 1030nm), ${F}_{\omega }^{(1)}={F}_{\omega }^{(2)}=0.0146$a.u., ${F}_{2\omega }^{(1)}/{F}_{\omega }^{(1)}={F}_{2\omega }^{(2)}/{F}_{\omega }^{(2)}=\sin (\alpha )$; 2*α* = 10^∘^ is the angle between the beams, the focal diameter is 200 *μ*m, and ${\phi }_{\omega ,2\omega }^{(1)}={\phi }_{\omega ,2\omega }^{(2)}$.

**Fig. 3:**
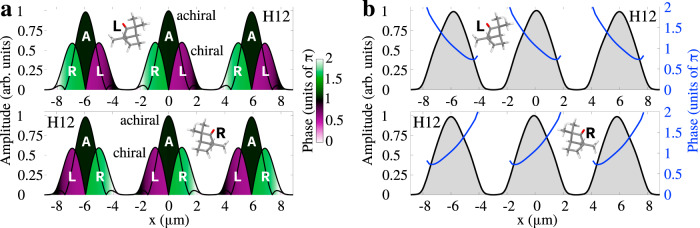
Enantio-sensitive nonlinear response of chiral matter to chirality-polarized light. **a** Amplitude of the achiral and chiral components of the nonlinear response driven by the space-time light structure presented in Fig. 2 in randomly oriented left-handed (upper panel) and right-handed (lower panel) fenchone molecules at frequency 12*ω*; the phase is encoded in the colors. **b** Amplitude (black) and phase (blue) of the total nonlinear response in the near field resulting from adding the achiral and chiral components of (**a**) for left-handed (upper panel) and right-handed (lower panel) fenchone. Laser parameters are the same as in Fig. 2 with ${\phi }_{\omega ,2\omega }^{(1)}={\phi }_{\omega ,2\omega }^{(2)}=0.44\pi$.

We now illustrate this general analysis with a specific example and demonstrate that racemic, chirality-polarized light can be used to induce the enantio-sensitive light bending and discriminate chiral molecules with high efficiency. Chirality-polarized light can be created using two beams propagating in the *xy* plane, at small angles *α* = ±5^∘^ with respect to the *y* axis, as shown in Fig. 2a. Both contain a fundamental field, linearly polarized in the plane of propagation, and a weak second harmonic component polarized orthogonal to this plane. In the overlap region, the total electric field is elliptically polarized in the *xy* plane at frequency *ω*, and it has a weak, linearly polarized, 2*ω* frequency component along *z*,

4 $${\bf{F}}(x,t)=\Re \left\{\right.\left[\right.{F}_{x}(x)\hat{{\bf{x}}}+i{F}_{y}(x)\hat{{\bf{y}}}\left]\right.{e}^{-i\omega t}+{F}_{z}(x){e}^{-2i(\omega t+\phi )}\hat{{\bf{z}}}\left\}\right.$$

where the two-color phase delay $\phi =\frac{{\phi }_{1}\;+\;{\phi }_{2}}{2}$, which controls the relative phase between the chiral and achiral responses, is determined by the two-color phase delays in the individual beams, *ϕ*_1_ and *ϕ*_2_ (see Methods). The spatial modulation of the three orthogonal polarization amplitudes, *F*_*x*_, *F*_*y*_ and *F*_*z*_, is described in Methods. The electric field vector, at a given point in space, draws a chiral Lissajous figure in **F**-space. Figure 2b shows that the field’s transverse spin ${{\bf{S}}}_{2\omega }\propto {{\bf{F}}}_{\omega }^{* }\times {{\bf{F}}}_{\omega }\propto {F}_{x}{F}_{y}\hat{{\bf{z}}}$ and ${{\bf{F}}}_{2\omega }={F}_{z}\hat{{\bf{z}}}$ change sign at different positions. As a result, the sign of their product **S**_2*ω*_ ⋅ **F**_2*ω*_, which determines the sign of light’s handedness, changes periodically in space (see Fig. 2c). This spatial distribution of the field’s handedness in the near field is recorded in the nonlinear response of the medium (see Fig. 3). In the lowest order of non-linearity, the strength of the local enantio-sensitive response is controlled by the chiral correlation function^26^ *h*^(5)^ ∝ **S**_2*ω*_ ⋅ **F**_2*ω*_∣**F**_*ω*_∣^2^ (see Methods) shown in Fig. 2c. We see a periodic structure, like that envisioned in Fig. 1d. The overall light field has mirror symmetry with respect to the *yz* plane (up to a global time shift) and is achiral. However, its handedness is polarized, with the *x*-component of the dipole of chirality (Eq. (2)) equal to:

5 $${\tilde{h}}_{x}\propto \cos ({\phi }_{2}-{\phi }_{1}){e}^{i({\phi }_{1}+{\phi }_{2})}$$

where *ϕ*_1_ and *ϕ*_2_ are the two-color phase delays in each of the two beams (see Fig. 2 and Methods). The difference *ϕ*_2_ − *ϕ*_1_ controls the amplitude of $\tilde{{\bf{h}}}$, which maximizes for *ϕ*_1_ = *ϕ*_2_. The phase of $\tilde{{\bf{h}}}$ is controlled by *ϕ*_1_ + *ϕ*_2_. This gives us control over the polarization of the field’s handedness: we set *ϕ*_1_ = *ϕ*_2_ to maximize its strength, and then vary *ϕ*_1_, *ϕ*_2_ synchronously to control the orientation of $\tilde{{\bf{h}}}$ in the complex plane. Note that the polarization of the field handedness in position space, evident in Fig. 2c, leads to a non-zero value of ∫*h*^(5)^*xdx*, which is proportional to ${\tilde{h}}_{x}$ for this definition of the unit cell (see Methods).

### Numerical results

We now quantify the interaction of the chirality-polarized light field in Fig. 2 with chiral matter. We consider a fundamental wavelength of 1030 nm with an intensity of 7.5 ⋅ 10^12^ W ⋅ cm^−2^ in each beam; the second harmonic intensity is 100 times weaker. As the two-color phase delays *ϕ*_1_ and *ϕ*_2_ control the spatial distribution of light’s handedness, they should remain constant across the interaction region. This condition can be fulfilled using a thin gas-jet target^27,28^. We assume randomly oriented chiral molecules in the gas phase, as in recent chiral HHG experiments^29,30^. In the liquid phase, one needs to ensure that the medium is sufficiently thin so dispersion does not modify significantly the two-color phase delays in the interaction region.

Figure 3 shows the nonlinear response of left- and right-handed randomly oriented fenchone molecules driven by this field at frequency 12*ω*, which is polarized along *z* (for other harmonics, see Supplementary Information). The total response (Fig. 3b) results from adding the contribution from the achiral and enantio-sensitive components (Fig. 3a) of emissions coming from all ‘slits’ (see Fig. 1 for two slits). Since the enantio-sensitive component of the response is *odd* with respect to *x*, the effect of exchanging the molecular enantiomer is equivalent to reversing the polarization of chirality of the field, which can be done by shifting the two-color phase delay in both beams (see Eq. (5)). The response at odd harmonic frequencies, shown in the Supplementary Information, is polarized along *x* and it is not enantio-sensitive.

Note that the single-molecule response, at a given point in space, can be enantio-sensitive in intensity because the driving field is locally chiral. However, since the overall light field is achiral, the total intensity signal, obtained after integration over *x*, is identical in left- and right-handed molecules. Still, the direction of polarization of the field’s handedness is imprinted on the phase of the nonlinear response, which depends strongly on the molecular handedness: the slope of the phase dependence on *x* is positive for right-handed molecules and negative in left-handed molecules, see Fig. 3b. The strength of this effect is controlled by the dipole of chirality ${\tilde{h}}_{x}$, which determines the deflection angle (see Methods).

**Fig. 4:**
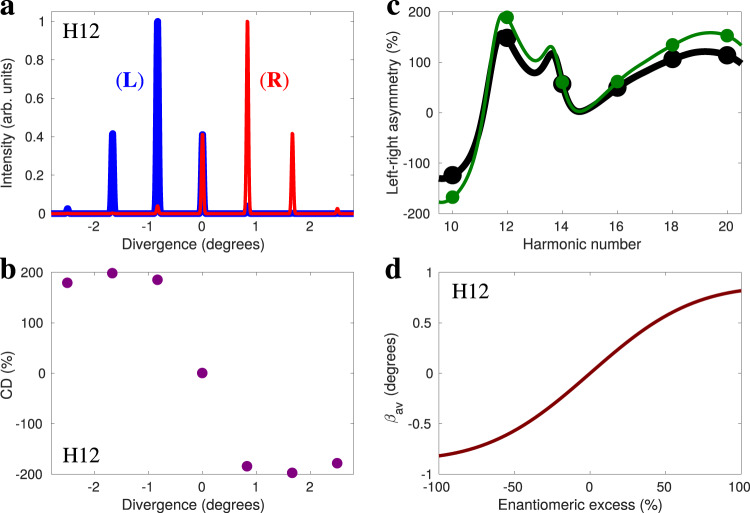
Enantio-sensitive light bending. **a** Far-field high-harmonic emission at frequency 12*ω* from left-handed (blue) and right-handed (red) randomly oriented fenchone molecules as a function of the emission angle. Field parameters are the same as in Fig. 3. **b** Chiral dichroism resolved on direction, $CD=2\frac{{I}_{L}-{I}_{R}}{{I}_{L}+{I}_{R}}$. **c** Left-right asymmetry in the macroscopic emission of even harmonics from 10 to 20, calculated including (black) or not including (green) the central emission peak. **d** Mean value of the emission angle as a function of the enantiomeric excess.

Figure 4a shows the harmonic 12 intensity in the far field. The total (angle-integrated) intensity is the same for left- and right-handed molecules, as in the near field (see Fig. 3b). However, the direction of emission is highly enantio-sensitive: while the left-handed molecules emit harmonics to the left (toward negative *x*), the right-handed molecules emit harmonics to the right (positive *x*). We control the enantio-sensitive direction of emission by controlling the polarization of the field’s handedness in our setup (see Eq. (5)). Figure 4b shows that chiral dichroism resolved in the emission angle, $CD(\beta )=2\frac{{I}_{L}(\beta )-{I}_{R}(\beta )}{{I}_{L}(\beta )+{I}_{R}(\beta )}$, reaches the ultimate efficiency limit of 200%. We find giant enantio-sensitivity in the direction of emission of all even harmonics (see Supplementary Information).

We can define the left-right asymmetry in the harmonic emission as $A=2\frac{I(\beta <0)-I(\beta > 0)}{I(\beta <0)+I(\beta > 0)}$, where *I* is the intensity of harmonic emission to the left (*β* < 0) and to the right (*β* > 0) from a medium of either left-handed (*I* = *I*_*L*_) or right-handed (*I* = *I*_*R*_) molecules. This angle-integrated quantity reaches very high values for all harmonic numbers, as shown in Fig. 4c. The direction of harmonic emission is correlated to the enantiomeric excess of the medium $ee=\frac{{C}_{R}-{C}_{L}}{{C}_{R}+{C}_{L}}$, with *C*_*R*_ and *C*_*L*_ being the concentrations of the right- and left-handed molecules, see Fig. 4d. The expectation value of the emission angle is then given by

6 $$\langle \beta \rangle =\frac{ee I_{1}}{I_{2}+e{e}^{2}I_{3}}$$

where $I_{1}$, $I_{2}$ and $I_{3}$ are angle-integrated quantities that do not depend on *ee* (see Methods). Eq. (6) allows us to quantify small values of enantiomeric excess in macroscopic mixtures (see Methods).

## Discussion

Polarization of chirality is a powerful concept which allows one to engineer highly efficient chiral interactions of racemic objects, taking advantage of the flexibility in shaping the local chirality of light. In particular, the spatial dependence of the field’s transverse spin ${{\bf{S}}}_{{\omega }_{n}}({\bf{r}})$ and the electric field component ${{\bf{F}}}_{{\omega }_{n}}({\bf{r}})$ parallel to it can be controlled separately. Here one can benefit from modern light shaping techniques including polarization shaping in space and time^31–33^. In contrast, in standard circularly polarized light, this opportunity is limited, since its electric and magnetic components are locked to each other. A non-zero dipole of chirality is present in any locally chiral field^26^ where the field’s transverse spin ${{\bf{S}}}_{{\omega }_{n}}({\bf{r}})$ and ${{\bf{F}}}_{{\omega }_{n}}({\bf{r}})$ have opposite parity.

Besides its application as a method for enantiomeric recognition, one can envision shaping light’s handedness and polarization so that they experience superoscillations^34^, creating sub-wavelength distribution of chiral and achiral emitters and enabling collective effects, such as sub-radiance, in chiral media^35^. The giant enantio-sensitivity to chirality polarized light may also be exploited to identify racemic aggregates of chiral matter exhibiting complex chirality patterns in space, and to quantify their degree of polarization of chirality. Finally, polarization of chirality can also be used for efficient separation of opposite enantiomers in racemic mixtures by extending the proposal of Ref. ^23^ to chirality polarized light, which would allow one to bypass the use of mechanical transition gratings and weak magnetic interactions.

## Methods

### Chiral diffraction by *N* slits

We start with diffraction by a single extended slit across which the phase varies linearly. This is the simplest extension of the double slit model introduced in the main text amenable for continuous distributions. In this case consider a dipole distribution of the form

7 $$P(x)={\mathcal{G}}(x){e}^{i(\xi \kappa x-\omega t)},$$

where ${\mathcal{G}}(x)\equiv | P(x)|$ and *κ* determines the linear variation of the phase across the slit. Note that this is equivalent to having an achiral response ${A}_{0}(x)={\mathcal{G}}(x)\cos (\kappa x)$ and a chiral response $\xi A(x){e}^{i\phi }=i\xi {\mathcal{G}}(x)\sin (\kappa x)$ as discussed in the main text. Under the usual approximations, the far field image associated to such dipole emission is given by

8 $$E(\theta )\propto \tilde{P}({k}_{x}){e}^{-i\omega t}=\tilde{{\mathcal{G}}}({k}_{x}-\xi \kappa ){e}^{-i\omega t},$$

where ${k}_{x}=k\sin \theta$, *k* = *ω*/*c*, *c* is the speed of light, *θ* is measured with respect to the perpendicular connecting the slit and the screen (as usual), and $\tilde{P}({k}_{x})$ and $\tilde{{\mathcal{G}}}({k}_{x})$ are the spatial Fourier transforms of *P*(*x*) and ${\mathcal{G}}(x)$, respectively. Equation (8) shows that the chiral contribution bends the far field image to positive or negative angles depending on the chirality *ξ* of the emitter. For small *θ*, the amount of bending is given by

9 $${{\Delta }}\theta \approx \xi \kappa /k.$$

In the case of *N* = 2*M* + 1 identical slits with their centers separated by a distance *L* we have

10 $$P(x)=\mathop{\sum }\limits_{n=-M}^{n=M}{P}_{0}(x-nL),\quad E(\theta )\propto \frac{\sin \left(N{k}_{x}L/2\right)}{\sin \left({k}_{x}L/2\right)}\tilde{{\mathcal{G}}}({k}_{x}-\xi \kappa ){e}^{-i\omega t}.$$

where *P*_0_ is given by Eq. (7). That is, we get the same result as for a single slit multiplied by the usual *N*-interference term that has peaks separated by *δθ* ≈ *λ*/*L* for small *θ* and *λ* = 2*π*/*k*. This *N*-chiral-slits model has a direct parallel in the non-collinear setup proposed in the main text and resulting in Fig. 4a. In that case the achiral and chiral responses can be modelled as ${P}^{ACH}(x) \sim {\mathcal{G}}(x){F}_{z}(x){e}^{i\phi }$ and ${P}^{CH}(x) \sim \xi {\mathcal{G}}(x){F}_{x}(x){F}_{y}(x)$, respectively, with ${\mathcal{G}}(x)$ a highly nonlinear function of the intensity, and the field amplitudes *F*_*i*_(*x*) given according to Eqs. (25)–(27). For a relative phase of *π*/4 between chiral and achiral responses, the corresponding replacements yield Δ*θ* ≈ *ξδθ* and *δθ* ≈ 2*α*/*j* for the *j*-th harmonic.

### Chirality dipoles and multipoles: a formal approach

Formally, the enantio-sensitive optical response to chiral light originates from the interference of two contributions to the light-induced polarization ${{\bf{P}}}_{\omega }={{\bf{P}}}_{\omega }^{ACH}+{{\bf{P}}}_{\omega }^{CH}$ at a given frequency *ω*, one of them not sensitive to chirality (${{\bf{P}}}_{\omega }^{ACH}$), and the other unique to chiral matter (${{\bf{P}}}_{\omega }^{CH}$), which is out of phase in media of opposite handedness. The microscopic intensity ∣**P**_*ω*_∣^2^ is sensitive to the interference of ${{\bf{P}}}_{\omega }^{CH}({\bf{r}})$ and ${{\bf{P}}}_{\omega }^{ACH}({\bf{r}})$ at the same point **r** through the term ${{\bf{P}}}_{\omega }^{ACH* }({\bf{r}})\cdot {{\bf{P}}}_{\omega }^{CH}({\bf{r}})+{\rm{c.c.}}$ This interference encodes the distributed handedness of light or matter (or both). That is, the near-field response records the distributed handedness locally. In contrast, the far-field signal provides access to the interference of the chiral and achiral contributions from the whole interaction region and is sensitive to spatial correlations of ${{\bf{P}}}_{\omega }^{ACH}({\bf{r}})$ and ${{\bf{P}}}_{\omega }^{CH}({\bf{r}}+{\bf{r}}^{\prime} )$ (see Methods for details). The far-field signal maps the distribution of handedness on observables such as the enantio-sensitive direction of light emission and the enantio-sensitive shape of the emission pattern on the screen. If the interaction region is thin, e.g. as usual in experiments with gas jets^27,28^, these observables are simply the different moments of the enantio-sensitive component of the intensity distribution in the reciprocal space, which is proportional to the real part of

11 $${\tilde{G}}_{\omega }({\bf{k}})\equiv {\tilde{{\bf{P}}}}_{\omega }^{ACH* }({\bf{k}})\cdot {\tilde{{\bf{P}}}}_{\omega }^{CH}({\bf{k}}).$$

(The subscript *ω* indicates that we consider far-field signals centered at frequency *ω* with bandwidth Δ*ω* ≪ *ω*, hence Δ*k* ≪ *k*; we omit this subscript below for brevity.) For example, the enantio-sensitive contributions to the total intensity and the average direction of emission are given by the zeroth and first moments of the distribution, respectively:

12 $$\langle{{\Delta }}{I}_{\omega }\rangle\propto \int {d}^{3}k\ \tilde{G}({\bf{k}})+\,{\text{c.c.}}\,,\,\,\,\,\langle{k}_{i}\rangle=\int {d}^{3}k\ {k}_{i}\tilde{G}({\bf{k}})+\,\text{c.c.}\,$$

The enantio-sensitive shape of the emission pattern on the screen is given by the higher moments:

13 $$\langle{k}_{i,j...}\rangle=\int {d}^{3}k\ {k}_{i}{k}_{j}...\tilde{G}({\bf{k}})+\,\text{c.c.}\,,\,\,\,$$

Eqs. (12)–(13) describe the multipoles of the enantio-sensitive intensity distribution in **k**-space. The different moments of $\tilde{G}({\bf{k}})$ reflect the fact that handedness may have non-trivial distributions both in coordinate and reciprocal space.

If the handedness of matter is distributed uniformly, then $\tilde{G}({\bf{k}})$ reflects the distributed handedness of light $\tilde{h}({\bf{k}})$, i.e. $\tilde{G}({\bf{k}})\propto \tilde{h}({\bf{k}})$ (see below for the definition of $\tilde{h}({\bf{k}})$). The enantio-sensitive contribution to light intensity, the direction of light emission, and the shape of the light spot on a screen will then encode zero, first, and higher order moments of $\tilde{h}({\bf{k}})$, respectively:

14 $${h}_{0}=\int {d}^{3}k\ ,\,\tilde{h}({\bf{k}}),\,\,\,\tilde{{\bf{h}}}=\int {d}^{3}k\,\, {\bf{k}}\tilde{h}({\bf{k}}),\,\,\,{\tilde{h}}_{ij...}=\int {d}^{3}k\,\, {k}_{i}{k}_{j}...\tilde{h}({\bf{k}}).$$

The enantio-sensitive contribution to the total intensity is only non-zero if light’s handedness is non-zero on average, *h*_0_ ≠ 0. But even if *h*_0_ = 0, enantio-sensitive effects remain. For example, if light is racemic, *h*_0_ = 0, but chirality polarized, i.e. $\tilde{{\bf{h}}}\;\ne\; 0$, we will see enantio-sensitive unidirectional deflection in the nonlinear optical response.

In general, the distributed handedness of racemic objects manifests itself in an entire array of tensorial enantio-sensitive observables. Their measurement requires acquisition of *N*-dimensional data sets, where *N* is the rank of the corresponding tensor.

### Spatial correlations of achiral and chiral responses

Here we show how the spatial correlations of chiral and achiral responses manifest in the far field observables. Rewriting Eq. (11) using the cross-correlation theorem, we find the relationship between the corresponding quantities in coordinate space:

15 $${G}_{\omega }({\bf{r}})=\int {d}^{3}{r}^{\prime}{{\bf{P}}}_{\omega }^{ACH* }({\bf{r}}^{\prime} )\cdot {{\bf{P}}}_{\omega }^{CH}({\bf{r}}^{\prime} +{\bf{r}}),$$

Using Eqs. (11) and (15), the Plancherel-Parseval identity, the property ${\mathcal{F}}\{\frac{{\partial }^{n}}{\partial {x}^{n}}f(x)\}(k)={(ik)}^{n}{\mathcal{F}}\{f(x)\}(k)$ for Fourier transforms ${\mathcal{F}}$, and the chain rule for partial derivatives, we get

16 $$\int {k}_{i}{k}_{j}\ldots {k}_{q}\tilde{G}({\bf{k}}){d}^{3}k 	={(-i)}^{n}\int {{\bf{P}}}^{ACH* }({{\bf{r}}}^{\prime})\cdot \frac{\partial }{\partial {r}_{i}^{\prime}}\frac{\partial }{\partial {r}_{j}^{\prime}}\ldots \frac{\partial }{\partial {r}_{q}^{\prime}}{{\bf{P}}}^{CH}({{\bf{r}}}^{\prime}){d}^{3}{r}^{\prime}\\ 	={(-i)}^{n}\int {{\bf{P}}}^{ACH* }({{\bf{r}}}^{\prime})\cdot \frac{\partial }{\partial {r}_{i}}\frac{\partial }{\partial {r}_{j}}\ldots \frac{\partial }{\partial {r}_{q}}{{\bf{P}}}^{CH}({{\bf{r}}}^{\prime}+{\bf{r}}){\left|\right.}_{{\bf{r}} = 0}{d}^{3}{r}^{\prime}\\ 	={(-i)}^{n}{(2\pi )}^{3/2}\frac{\partial }{\partial {r}_{i}}\frac{\partial }{\partial {r}_{j}}\ldots \frac{\partial }{\partial {r}_{q}}G({\bf{r}}){\left|\right.}_{{\bf{r}} = 0},$$

where *n* is the number of components of **k** multiplying $\tilde{G}({\bf{k}})$ on the left hand side.

Thus, the far-field enantio-sensitive observables can be generated using the spatial correlator *G*_*ω*_(**r**) of the achiral and chiral fields emitted by the chiral medium. For example, the enantio-sensitive contribution to the total intensity of the emitted light is proportional to the scalar $\Re \left\{{G}_{\omega }({\bf{r}}=0)\right\}$, the direction of emission is proportional to the vector $\Re \left\{i\nabla {G}_{\omega }({\bf{r}}){| }_{{\bf{r}} = 0}\right\}$, the shape of the emission pattern on the screen requires tensorial observables and is characterized by higher order derivatives such as $\Re \left\{i\frac{\partial }{\partial {r}_{i}}i\frac{\partial }{\partial {r}_{j}}{G}_{\omega }({\bf{r}}){| }_{{\bf{r}} = 0}\right\}$. Thus, the correlator *G*_*ω*_(**r**) and, in particular, its derivatives, presenting vectorial and tensorial quantities, reflect the distribution of relative handedness of light and matter across the interaction region. The vector $\Re \left\{i\nabla {G}_{\omega }({\bf{r}}){| }_{{\bf{r}} = 0}\right\}$ is sensitive to polarization of chirality, the higher order tensors encode chirality quadrupoles, octupoles and higher multipole moments.

### Connection between *G*_*ω*_(**r**), ${\tilde{G}}_{\omega }({\bf{k}})$ and light’s handedness in reciprocal space ${\tilde{h}}_{\omega }({\bf{k}})$

Let us consider the interaction of an optical field that has an arbitrary distribution of chirality with chiral matter whose handedness is uniform in space, e.g. an ensemble of randomly oriented chiral molecules. In this case, *G*_*ω*_(**r**), the coordinate space counterpart of ${\tilde{G}}_{\omega }({\bf{k}})$, gives us direct access to the distributed handedness of light. This statement is completely general. Here, we illustrate it using the lowest-order multiphoton processes that record the handedness of synthetic chiral light. In the lowest order, the achiral pathway is given by a linear response, and the chiral pathway is associated with an even-order nonlinear process^12,26^. For example, in a three-color field with frequencies *ω*_1_, *ω*_2_ and *ω*_3_ = *ω*_1_ + *ω*_2_, the linear response **P**^*ACH*^(**r**) at the frequency *ω*_3_ interferes with the second order response ${{\bf{P}}}_{{\omega }_{3}}^{CH}({\bf{r}})$ at the same frequency:

17 $${{\bf{P}}}_{{\omega }_{3}}^{ACH}({\bf{r}})={\chi }^{(1)}({\omega }_{3}){{\bf{F}}}_{{\omega }_{3}}({\bf{r}}),$$

18 $${{\bf{P}}}_{{\omega }_{3}}^{CH}({\bf{r}})={\chi }^{(2)}({\omega }_{3};{\omega }_{1},{\omega }_{2})\left[{{\bf{F}}}_{{\omega }_{1}}({\bf{r}})\times {{\bf{F}}}_{{\omega }_{2}}({\bf{r}})\right].$$

Here *χ*^(1)^(*ω*_3_) and *χ*^(2)^(*ω*_3_; *ω*_1_, *ω*_2_) are the linear and quadratic molecular susceptibilities averaged overall possible molecular orientations. The chiral response appears already in the dipole approximation^7,36^ and **r** enters as a parameter characterizing the spatial distribution of the field in the interaction region. The chiral response ${{\bf{P}}}_{{\omega }_{3}}^{CH}({\bf{r}})$ is proportional to the pseudoscalar handedness of matter *χ*^(2)^ and the pseudovector field ${{\bf{H}}}_{{\omega }_{1}+{\omega }_{2}}^{(2)}({\bf{r}})\equiv \left[{{\bf{F}}}_{{\omega }_{1}}({\bf{r}})\times {{\bf{F}}}_{{\omega }_{2}}({\bf{r}})\right]$ (see Ref. ^36^). (Note that in the case of the two-color chirality polarized field shown in Fig. 2c, the pseudovector field ${{\bf{H}}}_{2\omega }^{(2)}({\bf{r}})$ is proportional to the field’s spin ${{\bf{S}}}_{2\omega }\propto {{\bf{F}}}_{\omega }^{* }\times {{\bf{F}}}_{\omega }\propto {F}_{x}{F}_{y}\hat{{\bf{z}}}$, see Eq. (28) below).

If the handedness of matter is distributed uniformly, then ${G}_{{\omega }_{3}}({\bf{r}})$ reflects the distributed handedness of light: ${G}_{{\omega }_{3}}({\bf{r}})$ is sensitive to the spatial correlations between the components of its pseudovector field and electric field at frequency *ω*_3_ = *ω*_1_ + *ω*_2_:

19 $${G}_{{\omega }_{3}}({\bf{r}})\propto \int {d}^{3}{r}^{\prime}{{\bf{F}}}_{{\omega }_{3}}^{* }({\bf{r}}^{\prime} )\cdot {{\bf{H}}}_{{\omega }_{1}+{\omega }_{2}}({\bf{r}}^{\prime} +{\bf{r}}).$$

Its *k*-space counterpart ${\tilde{G}}_{{\omega }_{3}}({\bf{k}})$ is proportional to the light’s handedness at frequency *ω*_3_, ${\tilde{G}}_{{\omega }_{3}}({\bf{k}})\propto {\tilde{h}}_{{\omega }_{3}}({\bf{k}})$, which is given by the scalar product of its pseudovector field and electric field in *k*-space:

20 $${\tilde{h}}_{{\omega }_{3}}({\bf{k}})\equiv {\tilde{{\bf{H}}}}_{{\omega }_{1}+{\omega }_{2}}({\bf{k}})\cdot {\tilde{{\bf{F}}}}_{{\omega }_{3}}^{* }({\bf{k}}).$$

### Chirality-polarized light

Here we detail the non-collinear optical setup presented in Fig. 2. Our setup contains two non-collinear laser beams (*n* = 1, 2) that propagate in the *xy* plane, at small angles ± *α* with respect to the *y* direction.

21 $${{\bf{F}}}_{n}({\bf{r}},t)={e}^{-{\rho }_{n}^{2}/{\tilde{\omega }}^{2}}\ \Re \left\{\right.{F}_{\omega }{e}^{i({{\boldsymbol{\kappa }}}_{n}\cdot {\bf{r}}-\omega t)}{\hat{{\bf{e}}}}_{n}+{F}_{2\omega }{e}^{2i({{\boldsymbol{\kappa }}}_{n}\cdot {\bf{r}}-\omega t-{\phi }_{n})}\hat{{\bf{z}}}\left\}\right.,$$

where *ρ*_*n*_ is the radial distance to the beams’ axis, $\tilde{\omega }$ is the radial waist, ${{\boldsymbol{\kappa }}}_{1,2}=\kappa [{\pm}\!\sin (\alpha )\hat{{\bf{x}}}+\cos (\alpha )\hat{{\bf{y}}}]$ is the propagation direction, $\kappa =\frac{2\pi }{\lambda }$, *λ* is the fundamental wavelength, *ω* is the fundamental frequency, ${\hat{{\bf{e}}}}_{1,2}=\cos (\alpha )\hat{{\bf{x}}}\mp \sin (\alpha )\hat{{\bf{y}}}$ is the polarization vector of the fundamental field, and *ϕ*_*n*_ is the two-color phase delay. In the overlap region, *x* ≃ *ρ*_1_ ≃ *ρ*_2_ for small *α*, and the total electric field **F** = **F**_1_ + **F**_2_ can be written as

22 $${\bf{F}}({\bf{r}},t)={{\bf{F}}}_{\omega }\left(x,y\right){e}^{-i\omega t}+{{\bf{F}}}_{2\omega }\left(x,y\right){e}^{-2i\omega t}+{\rm{c.c.}},$$

where

23 $${{\bf{F}}}_{\omega }\left(x,y\right)={F}_{\omega }\left[{F}_{x}\left(x\right)\hat{x}-i{F}_{y}\left(x\right)\hat{y}\right]{e}^{i{\kappa }_{y}y},$$

24 $${{\bf{F}}}_{2\omega }\left(x,y\right)={F}_{2\omega }{F}_{z}\left(x\right)\hat{z}{e}^{2i\left({\kappa }_{y}y-{\phi }_{+}\right)},$$

${\kappa }_{x}=\kappa \sin \alpha$, ${\kappa }_{y}=\kappa \cos \alpha$, and ${\phi }_{\pm }=\frac{{\phi }_{2}\pm {\phi }_{1}}{2}$. That is, the total electric field is elliptically polarized in the *xy* plane at frequency *ω* and it has a linearly-polarized 2*ω* component along *z*. The *x* dependence of the field is given by

25 $${F}_{x}\left(x\right)=\cos \alpha \cos ({\kappa }_{x}x){e}^{-{\rho }_{n}^{2}/{\tilde{\omega }}^{2}},$$

26 $${F}_{y}\left(x\right)=\sin \alpha \sin ({\kappa }_{x}x){e}^{-{\rho }_{n}^{2}/{\tilde{\omega }}^{2}},$$

27 $${F}_{z}\left(x\right)=\cos (2{\kappa }_{x}x+2{\phi }_{-}){e}^{-{\rho }_{n}^{2}/{\tilde{\omega }}^{2}}.$$

### Moments of the field handedness relevant for the far field intensity

In general, the induced dipole produced by a field with frequencies *ω* and 2*ω* in an isotropic medium is given by^26^ ${{\bf{P}}}_{2\omega }={{\bf{P}}}_{2\omega }^{ACH}+{{\bf{P}}}_{2\omega }^{CH}$, where ${{\bf{P}}}_{2\omega }^{ACH}={\chi }^{(1)}{{\bf{F}}}_{2\omega }$ is the linear achiral response at frequency 2*ω*, ${{\bf{P}}}_{2\omega }^{CH}={\chi }^{(4)}{{\bf{H}}}_{2\omega }$ is the lowest order non-linear chiral response at frequency 2*ω*, and

28 $${{\bf{H}}}_{2\omega }=[{{\bf{F}}}_{\omega }^{* }\times {{\bf{F}}}_{\omega }][{{\bf{F}}}_{\omega }\cdot {{\bf{F}}}_{\omega }].$$

If the medium is homogeneous then the field (22) yields ${\tilde{G}}_{2\omega }({\bf{k}})={\chi }^{(1)* }{\chi }^{(4)}{\tilde{h}}_{2\omega }({\bf{k}})$ [cf. Eq. (11)], where ${\tilde{h}}_{2\omega }({\bf{k}})\equiv {\tilde{{\bf{F}}}}_{2\omega }^{* }({\bf{k}})\cdot {\tilde{{\bf{H}}}}_{2\omega }({\bf{k}})$ (see Eq. (20)) is the handedness of the reciprocal space form of the field. Using Eqs. (23)–(27) we obtain

29 $${\tilde{h}}_{2\omega }({\bf{k}})=C\left[\delta \left({k}_{x}+2{\kappa }_{x}\right){e}^{2i{\phi }_{-}}-\delta \left({k}_{x}-2{\kappa }_{x}\right){e}^{-2i{\phi }_{-}}\right]\delta ({k}_{y}-2{\kappa }_{y})\delta \left({k}_{z}\right),$$

where $C\equiv \frac{{F}_{2\omega }{F}_{\omega }^{4}}{{2}^{5}}{\left(2\pi \right)}^{3}\sin \left(4\alpha \right){e}^{2i{\phi }_{+}}$. (For a finite interaction region the infinitely narrow peaks represented by Dirac deltas widen accordingly). The overall phase of ${\tilde{h}}_{2\omega }({\bf{k}})$ is controlled by *ϕ*_+_ and its magnitude by the amplitudes of the fields *F*_*ω*_, *F*_2*ω*_, and the angle 2*α* between the beams. ${\tilde{h}}_{2\omega }({\bf{k}})$ has peaks at **k** = (±2*κ*_*x*_, 2*κ*_*y*_, 0). The asymmetry between these two peaks is controlled by the parameter *ϕ*_−_. The zeroth and first moments of ${\tilde{h}}_{2\omega }({\bf{k}})$ are given by [cf. Eq. (2)]

30 $${h}_{0}=2iC\sin \left(2{\phi }_{-}\right),$$

31 $$\tilde{{\bf{h}}}=4C\left(\right.\cos (2{\phi }_{-}),i\sin (2{\phi }_{-}),0\left)\right..$$

The effects of these moments on the far field intensity distribution follow from Eq. (12)

32 $$\langle {{\Delta }}{I}_{\omega }\rangle \propto \Re \left\{{\chi }^{\left(1\right)* }{\chi }^{\left(4\right)}{h}_{0}\right\}=-2\left|{\chi }^{\left(1\right)* }{\chi }^{\left(4\right)}C\right|\sin \left(2{\phi }_{-}\right)\sin \left(2{\phi }_{+}+{\phi }_{M}\right),$$

33 $$\langle {\bf{k}}\rangle 	=\Re \left\{{\chi }^{\left(1\right)* }{\chi }^{\left(4\right)}\tilde{{\bf{h}}}\right\}\\ 	=4\left|{\chi }^{\left(1\right)* }{\chi }^{\left(4\right)}C\right|\left[\cos \left(2{\phi }_{-}\right)\cos \left(2{\phi }_{+}+{\phi }_{M}\right),-\sin \left(2{\phi }_{-}\right)\sin \left(2{\phi }_{+}+{\phi }_{M}\right),0\right],$$

where *ϕ*_*M*_ is a molecular phase defined by ${\chi }^{\left(1\right)* }{\chi }^{\left(4\right)}=| {\chi }^{\left(1\right)* }{\chi }^{\left(4\right)}| {e}^{i{\phi }_{M}}$. Note that the *y* component of 〈**k**〉 simply reflects 〈Δ*I*_*ω*_〉 and that *ϕ*_−_ and *ϕ*_+_ control the molecule-independent and the molecule-dependent parts of the response, respectively.

### Moments of the field handedness relevant for the near field intensity

Analogously to how the enantio-sensitive contribution to the intensity in the far field is determined by the interference between ${\tilde{{\bf{P}}}}^{ACH}({\bf{k}})$ and ${\tilde{{\bf{P}}}}^{CH}({\bf{k}})$, the intensity in the near field is determined by the interference between **P**^*ACH*^(**r**) and **P**^*CH*^(**r**) at the same point in space. Such interference term is proportional to the chiral correlation function introduced in Ref. ^26^: ${h}_{2\omega }({\bf{r}})\equiv {{\bf{F}}}_{2\omega }^{* }({\bf{r}})\cdot {{\bf{H}}}_{2\omega }({\bf{r}})$. Note that, despite the notation, *h*_2*ω*_(**r**) is not the inverse Fourier transform of ${\tilde{h}}_{2\omega }({\bf{k}})$. For the field in Eq. (22) the lowest (fifth) order correlation function is given by

34 $${h}^{(5)}({\bf{r}}) 	= -\frac{i}{{2}^{4}}{F}_{\omega }^{4}{F}_{2\omega }\left\{\right.\sin \left(4\alpha \right)\left[\sin \left(4{\kappa }_{x}x+2{\phi }_{-}\right)-\sin \left(2{\phi }_{-}\right)\right]\\ 	\quad+\sin \left(2\alpha \right)\left[\sin \left(6{\kappa }_{x}x+2{\phi }_{-}\right)-\sin \left(2{\kappa }_{x}x-2{\phi }_{-}\right)\right]\left\}\right.{e}^{2i{\phi }_{+}}.$$

*h*^(5)^(**r**) is shown in Fig. 2d. Its zeroth moment of is equal to that of ${\tilde{h}}_{2\omega }({\bf{k}})$ in Eq. (30) because of the Plancherel-Parseval identity (see also Ref. ^26^). Its first moment for a single unit cell (see Fig. 2d) depends on the definition of the unit cell. For the central unit cell shown in Fig. 2d (three unit cells are shown) we get

35 $$\mathop{\int}\nolimits_{\!\!\!\!-{{\Delta }}x/2}^{{{\Delta }}x/2}x{h}^{(5)}(x)dx=-\frac{1}{{2}^{4}\left(3\pi \right)}i{F}_{\omega }^{4}{F}_{2\omega }{e}^{2i{\phi }_{+}}{({{\Delta }}x)}^{2}\cos \left(2{\phi }_{-}\right)$$

where ${{\Delta }}x=\lambda /(2\sin \alpha )$ and we assumed *α* ≪ 1. Thus, for such definition of the unit cell, the first moment of *h*^(5)^(*x*) has the same dependence on our control parameters *ϕ*_−_ and *ϕ*_+_ as the *x* component of $\tilde{{\bf{h}}}$.

### Calculation of high harmonic generation in fenchone

We have computed the high harmonic response of a gas-phase medium of randomly oriented left- and right-handed fenchone molecules using a similar procedure to that employed in Refs. ^37,38^. The dipole response in the near field (Fig. 3) is calculated by averaging over molecular orientations:

36 $${\bf{D}}(x,N\omega )=\iint {{\bf{D}}}_{{{\Omega }}\alpha }(x,N\omega )\ d{{\Omega }}\ d\alpha$$

where *x* is the near-field coordinate, *N* is the harmonic number, *ω* is the fundamental frequency, and **D**_Ω*α*_ is the dipole moment associated with a given molecular orientation in the frequency domain, which depends on the solid angle Ω and on the angle *α*. Integration over Ω and *α* was performed using the Lebedev quadrature^39^ of order 17 and the trapezoid method. The single-channel dipole response associated with each molecular orientation **D**_Ω*α*_ was evaluated using the saddle-point method, following the recipe described in^40^, which allows us to split **D**_Ω*α*_ in three terms:

37 $${{\bf{D}}}_{{{\Omega }}\alpha }(x,N\omega )={a}_{{{\Omega }}\alpha }^{ion}(x,N\omega )\cdot {a}_{{{\Omega }}\alpha }^{prop}(x,N\omega )\cdot {{\bf{a}}}_{{{\Omega }}\alpha }^{rec}(x,N\omega )$$

which describe strong-field ionization, propagation in the presence of the strong laser field, and radiative recombination. Let us briefly describe the procedure used for evaluating each term. The dependence on *x* and *Nω* is omitted for the sake of simplicity.

Strong-field ionization amplitudes are evaluated using the following expression:

38 $${a}_{{{\Omega }}\alpha }^{ion}= \left(\frac{2\pi }{i{\partial }^{2}S({t}_{r},{t}_{i},{\bf{p}})/\partial {t}_{i}^{2}}\right)^{1/2}{e}^{-iS(t^{\prime}_{i} ,{t}_{i},{\bf{p}})}{\mathcal{F}}\{{{{\Psi }}}_{i}\}(\Re \{{\bf{k}}(t^{\prime}_{i} )\})$$

where ${t}_{i}=t_{i}^{\prime} +it_{i}^{\prime\prime}$ is the complex ionization time, **p** is the canonical momentum, which is related to the kinetic momentum via **k**(*t*) = **p** + **A**(*t*), here **A**(*t*) is the vector potential: **F**(*t*) = − ∂**A**(*t*)/∂*t*, ${\mathcal{F}}\{{{{\Psi }}}_{i}\}$ is the Fourier transform of the initial state wave function Ψ_*i*_, and

39 $$S(t,t^{\prime} ,{\bf{p}})=\frac{1}{2}\mathop{\int}\nolimits_{\!\!\!\!t^{\prime} }^{t}d\tau \left[\right.{\bf{p}}+{\bf{A}}(\tau ){\left]\right.}^{2}+Ip(t-t^{\prime} )$$

where *Ip* is the ionization potential.

Electron propagation in the continuum is described via

40 $${a}_{{{\Omega }}\alpha }^{prop}=\left(\frac{2\pi }{i({t}_{r}-{t}_{i})}\right)^{3/2}{e}^{-iS(t^{\prime}_{r} ,t^{\prime}_{i} ,{\bf{p}})}$$

where ${t}_{r}=t_{r}^{\prime} +it_{r}^{\prime\prime}$ is the complex recombination time.

Recombination amplitudes are given by

41 $${{\bf{a}}}_{{{\Omega }}\alpha }^{rec}=\left(\frac{2\pi }{i{\partial }^{2}S({t}_{r},{t}_{i},{\bf{p}})/\partial {t}_{r}^{2}}\right)^{1/2}{e}^{-iS({t}_{r},t^{\prime}_{r} ,{\bf{p}})+iN\omega {t}_{r}}{{\bf{d}}}_{{{\Omega }}\alpha }^{rec}\left(\right.{\bf{k}}(t^{\prime}_{r} )\left)\right.$$

where ${{\bf{d}}}_{{{\Omega }}\alpha }^{rec}$ is the photo-recombination matrix element, computed numerically using the static-exchange density functional theory (DFT) method^37,41–45^.

The intensity of macroscopic harmonic emission in the far field (Fig. 4) is calculated from the near-field response of the medium (Eq. (36)) using the Fraunhofer diffraction equation:

42 $${\bf{I}}(\beta ,N\omega )\propto {(N\omega )}^{4}\left|\mathop{\int}\nolimits_{\!\!\!\!-\infty }^{\infty }{\bf{D}}(x,N\omega ){e}^{-i\frac{N\omega }{c}x\beta }dx\right|^{2}$$

where *β* is the divergence, i.e. the angle of emission with respect to the *y* axis, and *c* is the speed of light. The mechanism responsible for the strong enantio-sensitivity in the direction of light emission is general and independent of the particular molecular system. Thus, one should expect to find similarly strong enantio-sensitivity in other chiral molecules, and also when computed using different theoretical approaches.

### Relation between the emission angle and the enantiomeric excess

The *z*-polarized component of the (even) harmonic radiation in a given point in the far field, for a given harmonic number, is given by

43 $${F}_{z}(ee,\beta )={F}_{2\omega }{F}_{a}(\beta )+\frac{1+ee}{2}\alpha {F}_{R}(\beta )+\frac{1-ee}{2}\alpha {F}_{L}(\beta )={F}_{2\omega }{F}_{a}(\beta )+ee\ \alpha \ {F}_{R}(\beta )$$

where $ee=\frac{{C}_{R}-{C}_{L}}{{C}_{R}+{C}_{L}}$ is the enantiomeric excess, *C*_*R*_ and *C*_*L*_ being the concentrations of left and right-handed molecules, the achiral contribution *F*_2*ω*_*F*_*a*_ depends linearly on *F*_2*ω*_, and the chiral contribution from left- and right-handed molecules *αF*_*R*_ and *αF*_*L*_ (*F*_*R*_ = −*F*_*L*_) depend linearly on *α*, for weak *F*_2*ω*_ and *α*. The harmonic intensity in a given point in the far field is thus given by

44 $$I(ee,\beta )={F}_{2\omega }^{2}{I}_{a}(\beta )+ee\ \alpha {F}_{2\omega }{I}_{aR}(\beta )+e{e}^{2}\ {\alpha }^{2}{I}_{R}(\beta )$$

where *I*_*a*_(*β*) = ∣*F*_*a*_(*β*)∣^2^, *I*_*R*_(*β*) = ∣*F*_*R*_(*β*)∣^2^, ${I}_{aR}(\beta )={F}_{a}^{* }(\beta ){F}_{R}(\beta )+{F}_{a}(\beta ){F}_{R}^{* }(\beta )$. These quantities do not depend on *α* or *F*_2*ω*_, for weak values of these parameters. Let us calculate the expectation value of the emission angle, given by

45 $$\langle \beta \rangle =\frac{\int I(ee,\beta )\beta d\beta }{\int I(ee,\beta )d\beta }$$

Inserting Eq. (44) into Eq. (45), and taking into account that *I*_*a*_ and *I*_*R*_ are even functions with respect to *β*, and that *I*_*aR*_ is odd, we obtain

46 $$\langle \beta \rangle =\frac{ee\ \alpha {F}_{2\omega }{\tilde{I}}_{aR}}{{F}_{2\omega }^{2}{\tilde{I}}_{a}+e{e}^{2}\ {\alpha }^{2}{\tilde{I}}_{R}}$$

where

47 $${\tilde{I}}_{aR}=\int {I}_{aR}(\beta) \beta d\beta$$

48 $${\tilde{I}}_{a} =\int {I}_{a}(\beta) d\beta$$

49 $${\tilde{I}}_{R}=\int {I}_{R}(\beta) d\beta$$

These angle-integrated quantities do not depend on *ee*, *α* or *F*_2*ω*_. Eq. (46) allows us to quantify *ee* in mixtures of opposite enantiomers. It also allows us reconstruct the values of ${\tilde{I}}_{aR}$, ${\tilde{I}}_{a}$ and ${\tilde{I}}_{R}$ by measuring 〈*β*〉 in mixtures of different *ee*. For small values of *ee*, where the determination of this quantity is usually more challenging, we have

50 $$\langle \beta \rangle \simeq \frac{ee\ \alpha {\tilde{I}}_{aR}}{{F}_{2\omega }{\tilde{I}}_{a}}$$

which allows us to determine *ee* by measuring 〈*β*〉 for different values of *F*_2*ω*_.

## Supplementary information

Supplementary Information

## Data Availability

The data that support the plots within this paper and other findings of this study are available from the corresponding authors upon reasonable request. Correspondence should be addressed to david.ayuso@mbi-berlin.de, andres.ordonez@mbi-berlin.de and olga.smirnova@mbi-berlin.de.
