# Supplementary material for: Enantio-sensitive unidirectional light bending
Source: Nat Commun. 2021 Jun 24;12:3951. doi: 10.1038/s41467-021-24118-4 (PMC8225816; doi:10.1038/s41467-021-24118-4)
Supplement: Supplementary file 1 — Supplementary Information [file 41467_2021_24118_MOESM1_ESM.pdf]

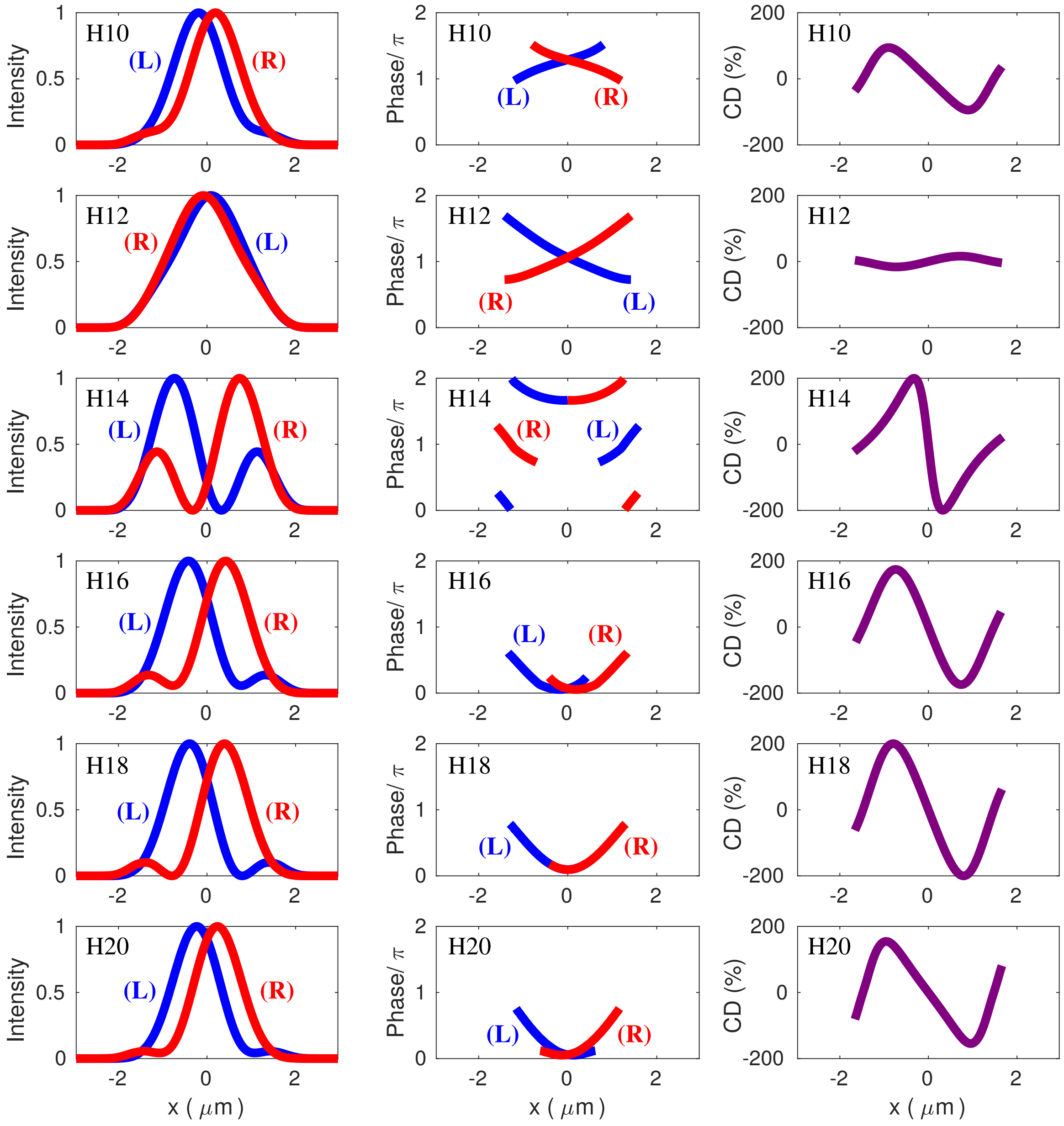

Supplementary Figure 1: **Enantio-sensitive HHG in the near field.** Normalized intensity (left panels) and phase (central panels) of the z-polarized high harmonic response driven by one unit cell of our chirality-polarized light field (Fig. 2 of main text) in randomly oriented left- (blue) and right-handed (red) fenchone molecules, and spatially-resolved chiral dichroism (right panels), $CD(x) = 2\frac{I_L(x)-I_R(x)}{I_L(x)+I_R(x)}$.

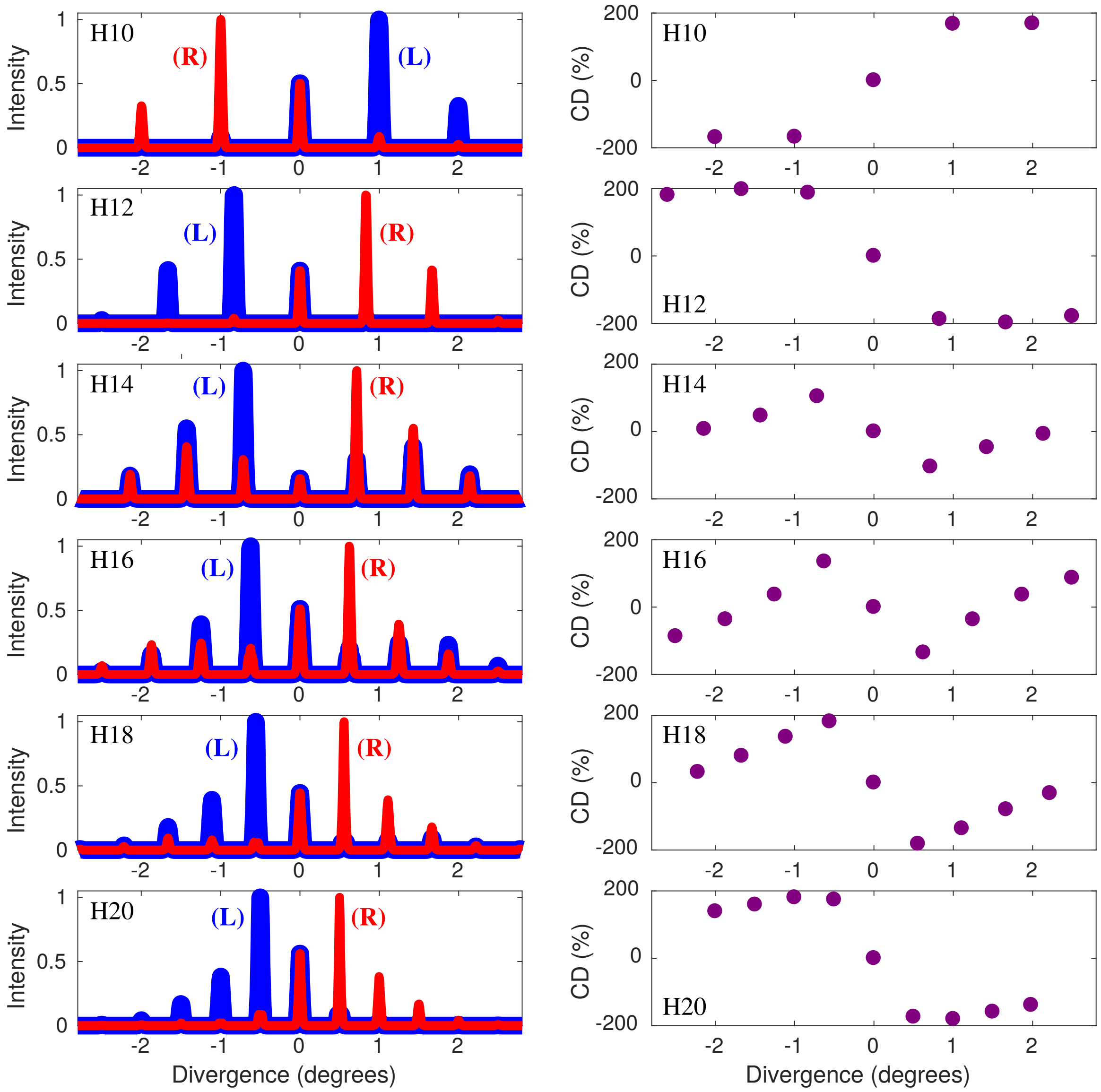

Supplementary Figure 2: **Enantio-sensitive HHG in the far field.** Normalized intensity of the z-polarized high harmonic emission from randomly oriented left-handed (blue) and right-handed (red) fenchone molecules in the far field as a function of the emission angle β (left panels) and angularly-resolved chiral dichroism (right panels), $CD(\beta) = 2\frac{I_L(\beta)-I_R(\beta)}{I_L(\beta)+I_R(\beta)}$.

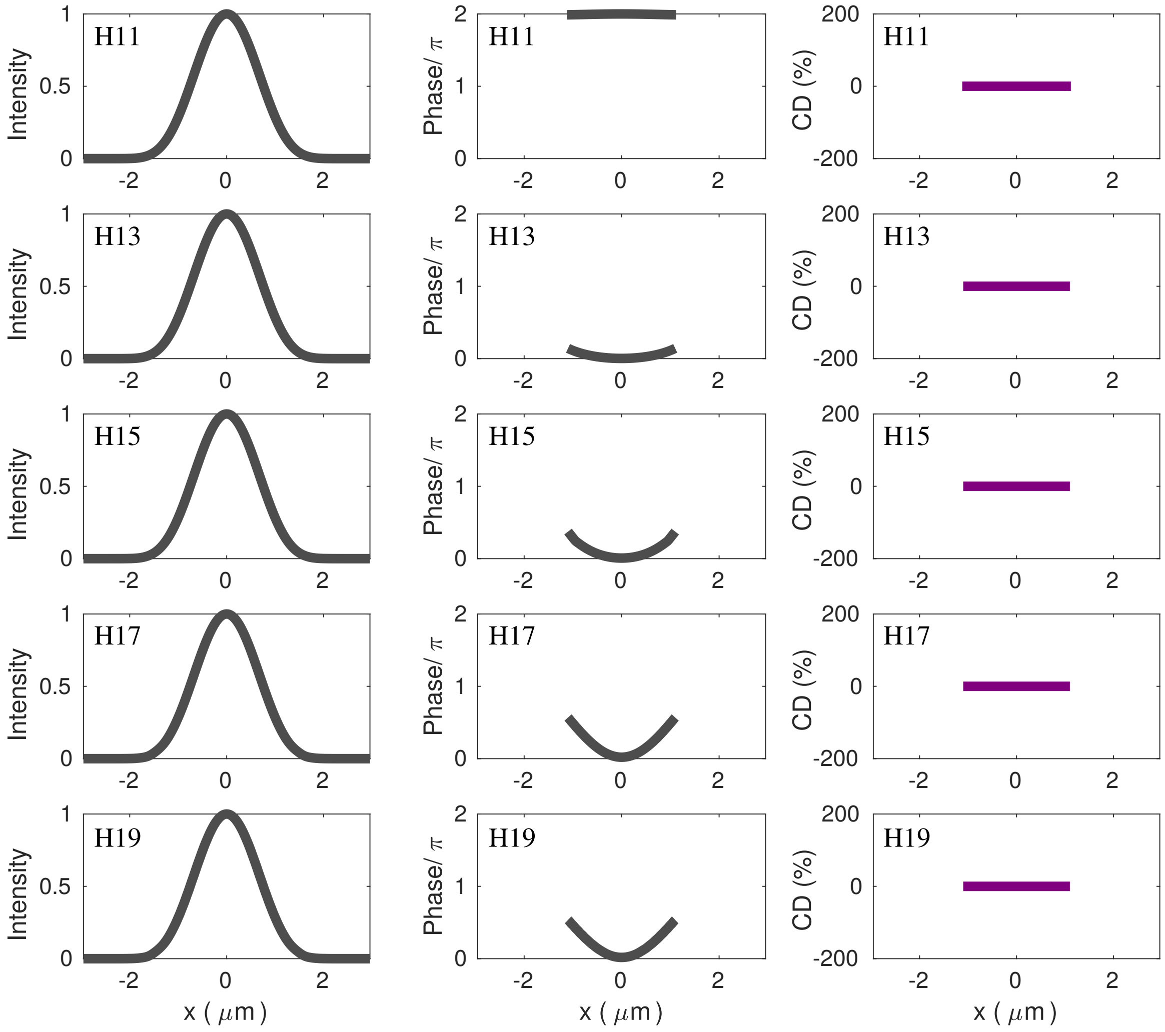

Supplementary Figure 3: **Non-enantio-sensitive HHG in the near field.** Same as Fig. 1, for the x-polarized odd harmonics. The response of opposite molecular enantiomers is identical (left and central panels), and thus the spatially-resolved chiral dichroism is zero (right panels).

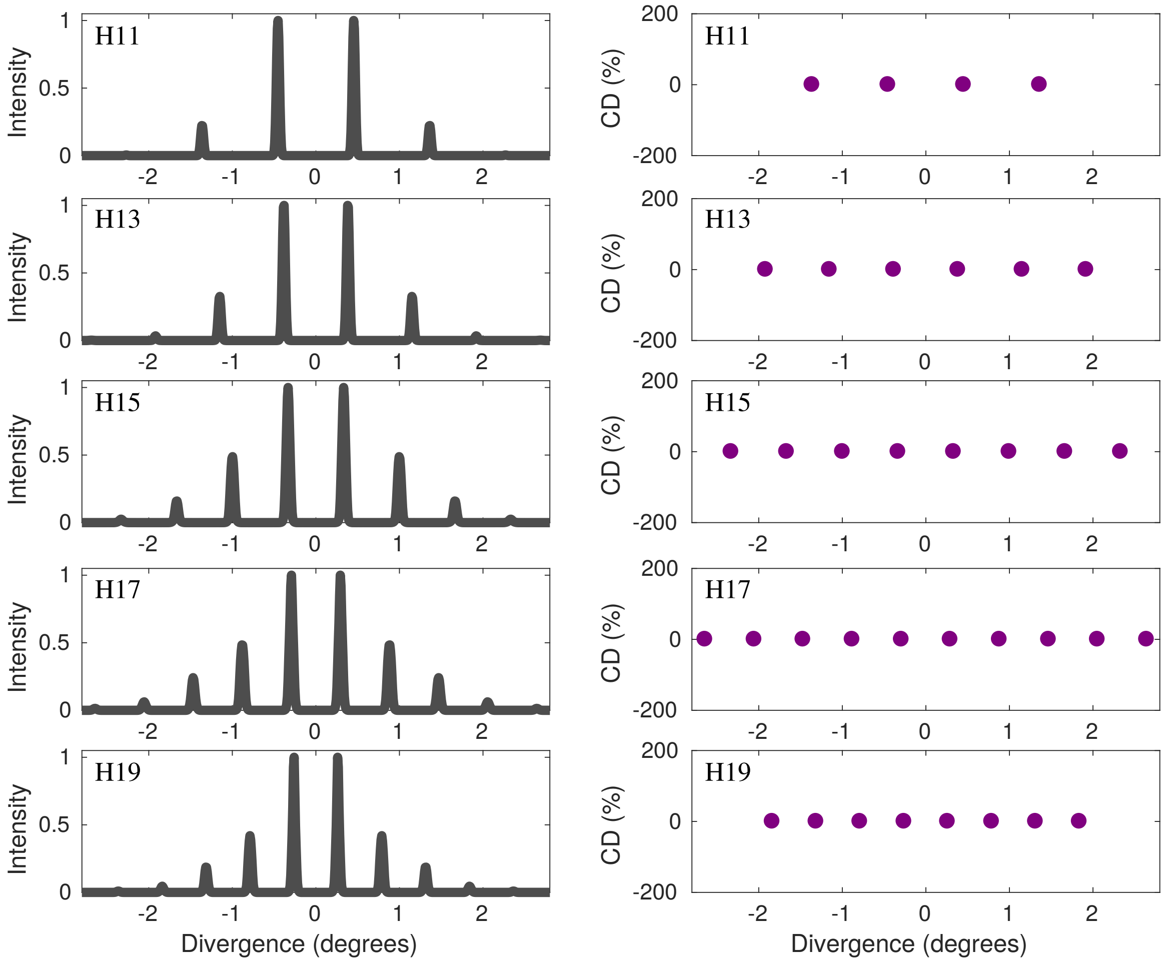

Supplementary Figure 4: **Non-enantio-sensitive HHG in the far field.** Same as Fig. 2, for the x-polarized odd harmonics. The response of opposite molecular enantiomers is identical (left and central panels), and thus the angularly-resolved chiral dichroism is zero (right panels).
